# RASGRP1 Deficiency Manifesting as Severe Vasculopathy and Fatal Autoimmune Hemolytic Anemia

**DOI:** 10.1002/jha2.70275

**Published:** 2026-05-29

**Authors:** Kosar Asna Ashari, Bahareh Yaghmaie, Neda Pak, Mohammad Shahrooei, Vahid Ziaee, Nima Parvaneh

**Affiliations:** ^1^ Department of Pediatrics Tehran University of Medical Sciences Tehran Iran; ^2^ Children's Medical Center Pediatrics Center of Excellence Tehran Iran; ^3^ Pediatric Rheumatology Research Group Rheumatology Research Center Tehran University of Medical Sciences Tehran Iran; ^4^ Pediatric Rheumatology Society of Iran Tehran Iran; ^5^ Division of Pediatric Intensive Care Department of Pediatrics Children's Medical Center Tehran University of Medical Sciences Tehran Iran; ^6^ Dr. Shahrooei Lab Tehran Iran

**Keywords:** autoimmunity, EBV, inborn error of immunity, lymphoproliferation, RASGRP1, vasculopathy

## Abstract

**Background:**

RASGRP1 deficiency is a rare inborn error of immunity characterized by immunodeficiency, autoimmunity, and lymphoproliferation.

**Results:**

We report a 5‐year‐old male with novel homozygous splice‐donor mutations in *RASGRP1*(c.1720+1G>A and c.1720+2T>C) who presented with severe vasculopathy (ischemic stroke and thrombosis), secondary antiphospholipid syndrome, and fatal refractory autoimmune hemolytic anemia.

**Conclusion:**

A review of 14 previously reported cases (plus current case) confirms that while infections (100%) and lymphoproliferation (87%) are common, vascular autoimmunity is an emerging life‐threatening phenotype. Hematopoietic stem cell transplantation remains the only curative therapy, as conservative management carries high mortality. Early genetic diagnosis is essential for optimal management.

**Trial Registration**: The authors have confirmed clinical trial registration is not needed for this submission

**Abbreviations**
AIHA: autoimmune hemolytic anemia 
CMV: cytomegalovirus
DVT
: deep venous thrombosis 
EBV: Epstein–Barr virus 
ESR: erythrocyte sedimentation rate
HLH: hemophagocytic lymphohistiocytosis 
HSCT: hematopoietic stem cell transplantation 
ITP: immune thrombocytopenic purpura 
IVIG: intravenous immunoglobulin
MAPK: mitogen‐activated protein kinase 
MCA: middle cerebral artery 
MRA: magnetic resonance angiography
MRI: magnetic resonance imaging 
RASGRP1: RAS guanyl‐releasing protein 1 
SLE: systemic lupus erythematosus
TTP: thrombotic thrombocytopenic purpura 
VZV: varicella‐zoster virus
WES: whole‐exome sequencing 
XLP: X‐linked lymphoproliferative disorder

## 1 Introduction

RAS guanyl‐releasing protein 1 (RASGRP1) is a critical guanine nucleotide exchange factor that activates the RAS‐MAPK pathway, which is essential for lymphocyte development and T‐cell receptor (TCR) signaling [1, 2]. Since 2016, biallelic *RASGRP1* mutations have been recognized as a cause of a distinct inborn error of immunity (IEI) often mimicking autoimmune lymphoproliferative syndrome (ALPS) [3]. While the classic triad includes immunodeficiency, autoimmunity, and Epstein–Barr virus (EBV)‐induced lymphoproliferation, the full clinical spectrum is still emerging. Specifically, vascular complications and life‐threatening refractory autoimmunity have been under‐reported. We present a fatal case of RASGRP1 deficiency characterized by large‐vessel vasculopathy and fatal autoimmune hemolytic anemia (AIHA), followed by a synthesis of all 15 known cases in the literature to guide clinical recognition [3, 4, 5, 6, 7, 8, 9, 10, 11].

## 2 Results

### 2.1 Case Presentation

A 5‐year‐old male, the first child of consanguineous Iranian parents, was referred after experiencing a series of severe inflammatory and infectious events. His medical history commenced in early infancy with a generalized bullous pemphigoid‐like eruption requiring intermittent topical steroids. At the age of 4, he endured an exceptionally severe varicella‐zoster virus (VZV) infection that required extended hospitalization.

**FIGURE 1:**
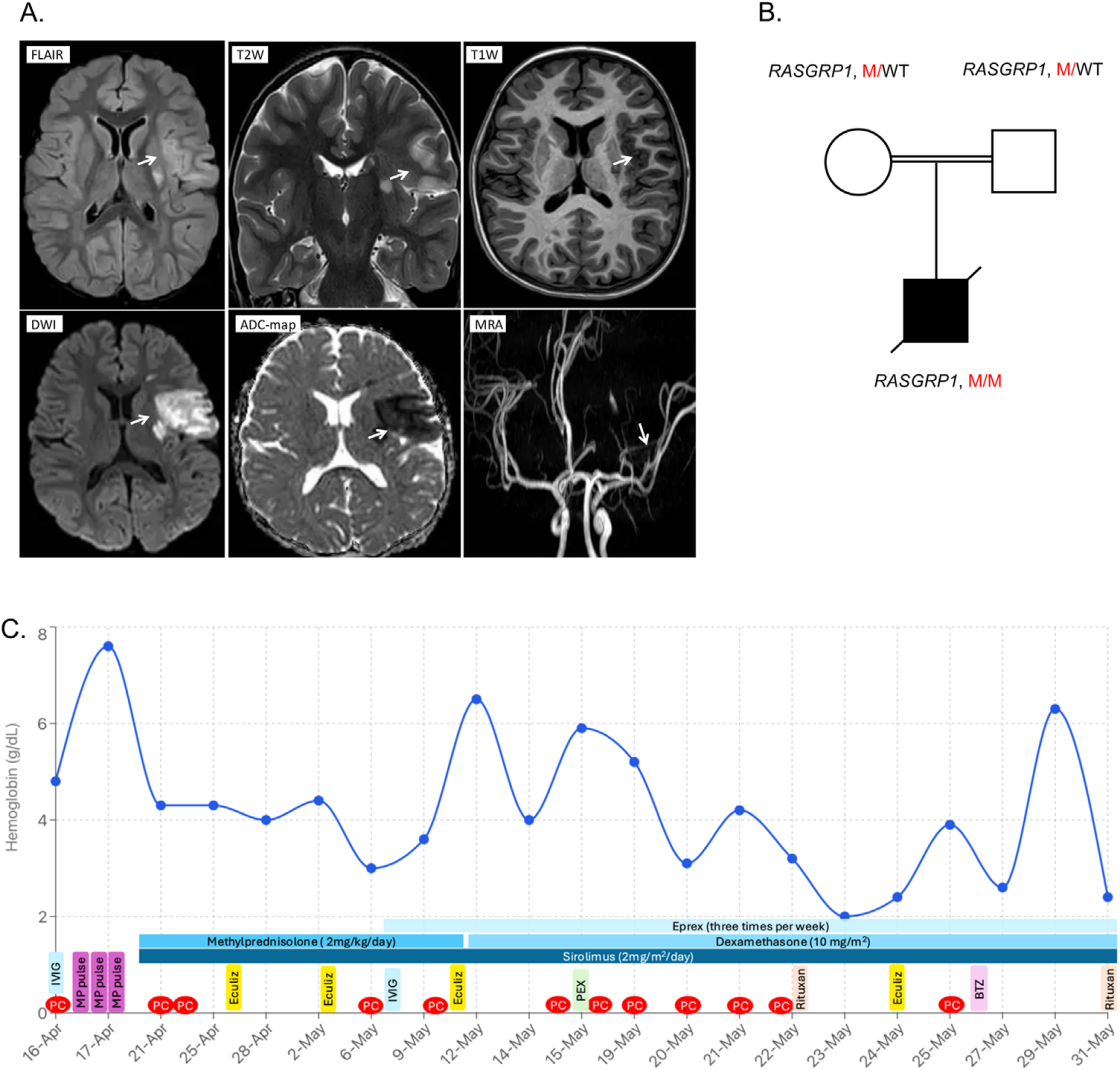
Neuroimaging and laboratory features of the patient with RASGRP1 deficiency. (A) Brain MRI revealed abnormal signal intensity in the posterior aspect of left putamen, cortex, and subcortical white matter of left insula and inferior frontal gyrus, hypersignal on FLAIR and T2W sequences and hypo‐signal on T1W images (white arrows), and the affected areas showed restriction on DWI and ADC‐map sequences compatible with acute infarction in left middle cerebral artery (MCA) vascular territory. Brain MRA demonstrated tapering and complete occlusion of one of the M2 branches of left MCA (white arrows). Note the paucity of distal branches of the left MCA compared with contralateral side. (B) Familial segregation of the c.1720+1G>A and c.1720+2T>C *RASGRP1* mutations in the index case and his parents. M, mutant; WT, wild type. (C) Longitudinal hemoglobin levels during the second admission demonstrating refractory hemolytic anemia. ADC, apparent diffusion coefficient; DWI, diffusion‐weighted imaging; FLAIR, fluid‐attenuated inversion recovery; MRI, magnetic resonance imaging; T1W, T1 weighted; T2W, T2 weighted.

The first admission was prompted by the abrupt onset of acute aphasia and right‐sided hemiplegia. Brain magnetic resonance imaging (MRI) and MR angiography (MRA) confirmed an acute ischemic infarction involving the territory of the left middle cerebral artery (MCA) (Figure 1A). During the initial stabilization phase, the clinical trajectory was further complicated by the development of deep venous thrombosis (DVT) in the left lower limb and an episode of pulmonary hemorrhage, indicating a systemic prothrombotic and inflammatory condition.

Laboratory investigations indicated severe pan‐T‐cell lymphopenia, evidenced by a CD3+ count of 460 cells/µL and a critically low CD4+ T‐cell count of 118 cells/µL. He also displayed profound pancytopenia and markedly elevated inflammatory markers. A comprehensive autoimmune panel revealed significantly positive results for lupus anticoagulant and elevated titers of anti‐beta‐2 glycoprotein and anti‐cardiolipin antibodies, supporting a diagnosis of secondary antiphospholipid syndrome (APS) (Table S1).

Whole‐exome sequencing (WES) identified two adjacent homozygous splice‐donor variants in *RASGRP1* (c.1720+1G>A and c.1720+2T>C in *cis* state) impacting the invariant GT dinucleotide at the donor site of intron 13 (NM_005739.4) (Figure 1B). In silico analysis using SpliceAI, Human Splicing Finder, and MaxEntScan anticipated to induce abnormal splicing (e.g., exon skipping or intron retention), resulting in a truncated or non‐functional protein. Six weeks later, the patient was readmitted with life‐threatening, Coombs‐positive AIHA. The hemolysis proved remarkably refractory to standard care. Despite aggressive multimodal rescue therapy consisting of high‐dose methylprednisolone, intravenous immunoglobulin (IVIG), eculizumab, sirolimus, bortezomib, and plasmapheresis, the patient's condition continued to deteriorate (Figure 1C). He ultimately succumbed to progressive anemia and sudden cardiac arrest 45 days after the second admission.

### 2.2 Review of Reported Cases

**FIGURE 2:**
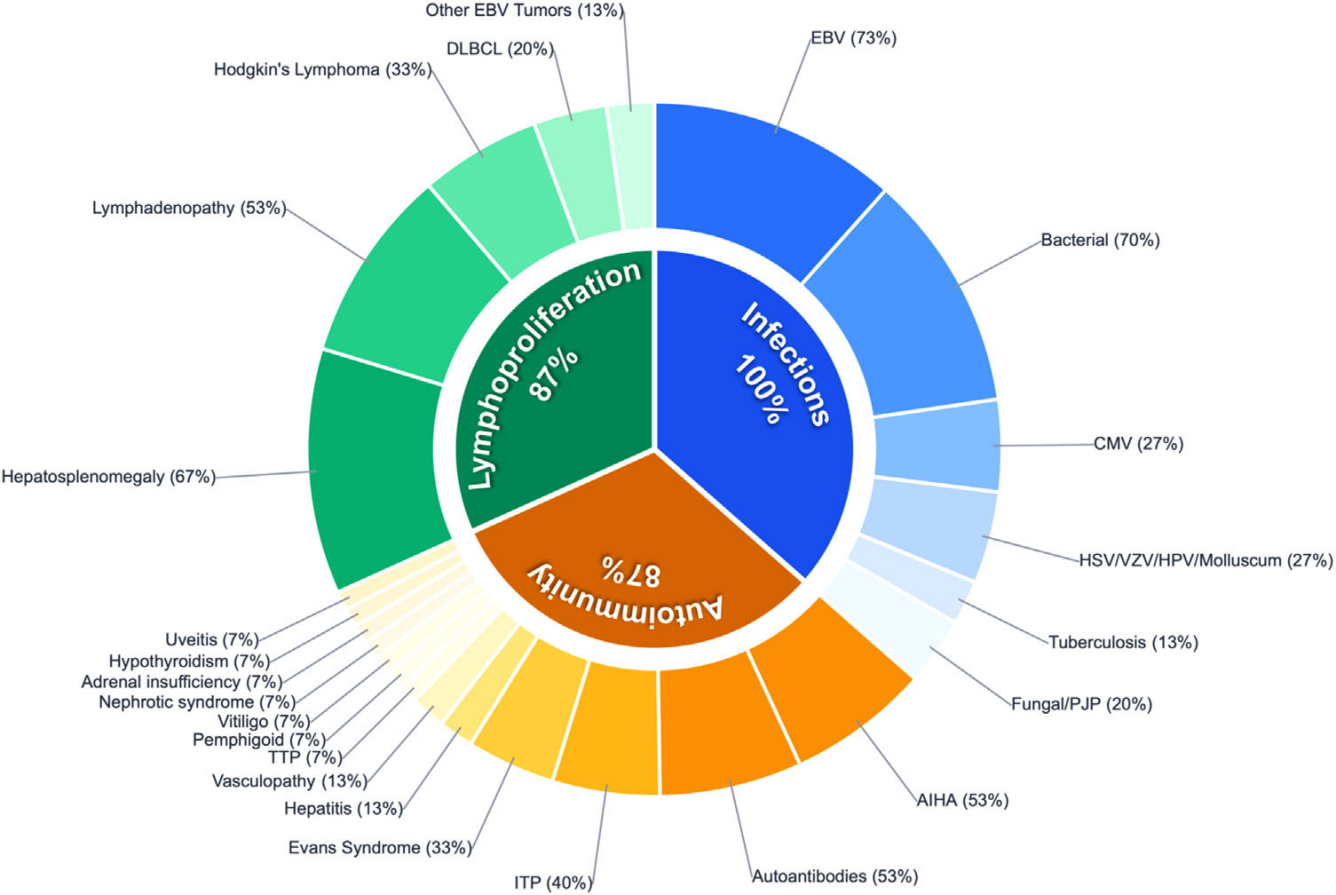
Clinical manifestations of RASGRP1‐deficient patients according to our review. AIHA, autoimmune hemolytic anemia; DLBCL, diffuse large B‐cell lymphoma; EBV, Epstein–Barr virus; ITP, immune thrombocytopenic purpura; TTP, thrombotic thrombocytopenic purpura.

A comprehensive analysis of the 14 previously reported cases alongside our index patient (P15) shows a notably consistent yet evolving clinical landscape for biallelic RASGRP1 deficiency [3, 4, 5, 6, 7, 8, 9, 10, 11] (Figure 2, Table S2). The cohort consists of 15 patients from 13 unrelated families, primarily of consanguineous Middle Eastern or North African origin, with a median age of symptom onset of 12 months. The range of mutations encompasses missense, nonsense, and splice‐site variants, with truncating mutations often associated with a more severe clinical course in early life.

Recurrent infections represent a universal feature (100%) of this disease. While bacterial sinopulmonary infections are prevalent, with a notable susceptibility to viral pathogens, particularly EBV in 73% of cases and cytomegalovirus (CMV) in 27%. This viral vulnerability is intrinsically associated with the high incidence of lymphoproliferative disease (87%). Patients frequently endure splenomegaly and lymphadenopathy, which often advance to high‐grade malignancies. Significantly, nearly 40% of the reported patients developed B‐cell lymphomas, including Hodgkin lymphoma (HL) and diffuse large B‐cell lymphoma (DLBCL), so underscoring the role of RASGRP1 in controlling EBV‐driven B‐cell transformation.

Immune dysregulation and autoimmunity were identified in 87% of the patients, typically manifesting as refractory cytopenias such as AIHA and immune thrombocytopenic purpura (ITP). Our index case (P15) significantly broadens this spectrum by introducing severe large‐vessel vasculopathy and secondary APS as life‐threatening manifestations. This suggests that the loss of RASGRP1‐mediated signaling impairs not only T‐cell‐dependent viral clearance but also B‐cell tolerance, leading to the production of diverse autoantibodies.

Managing RASGRP1 deficiency is a significant clinical challenge. Our review highlights a significant divergence in outcomes based on treatment modality. Four patients received hematopoietic stem cell transplantation (HSCT), all of whom achieved full clinical remission and are alive. Conversely, individuals managed conservatively with immunosuppressive agents (including steroids, rituximab, and sirolimus) faced a mortality rate of roughly 45%. Deaths were primarily ascribed to refractory AIHA, thrombotic thrombocytopenic purpura (TTP), or complications of lymphoma. This data highlights that although conservative approaches may provide temporary control, HSCT is currently the only curative intervention capable of halting the combined immunodeficiency, lymphomagenesis, and fatal autoimmunity linked with this defect.

## 3 Discussion

RASGRP1 deficiency is a rare IEI characterized by impaired TCR signaling, leading to a complex phenotype of immune dysregulation and combined immunodeficiency [3, 11]. Our analysis of the 15 cases documented to date corroborates a clinical triad of early‐onset immune dysregulation, universal susceptibility to infections (100%), and a high risk of EBV‐associated malignancies (53%).

Although the phenotype substantially overlaps with ALPS, several distinctions exist. Patients with classical ALPS typically generally have an increase in double‐negative T (DNT) cells and a comparatively low incidence of severe infections [12]. Conversely, individuals with RASGRP1 deficiency typically show normal DNT counts but suffer from life‐threatening bacterial and viral infections [3]. Furthermore, the oncogenic profile of this condition mirrors “narrow‐spectrum” EBV‐susceptible IEIs, such as CD27, CD70 or ITK deficiencies, rather than the broad lymphoproliferation seen in ALPS [13, 14].

Unlike X‐linked lymphoproliferative disease 1 (XLP1), which is defined by hemophagocytic lymphohistiocytosis (35%) and dysgammaglobulinemia (51%) [15], RASGRP1 deficiency exhibits a much higher penetrance of autoimmunity (87%).

A landmark finding in our patient (P15) is the presentation of severe large‐vessel vasculopathy, manifesting as ischemic stroke and DVT. Although refractory cytopenias such as AIHA and ITP are well‐documented [6, 10], the identification of lupus anticoagulant and anti‐beta‐2 glycoprotein antibodies substantiates that the autoimmune spectrum extends to secondary APS. This indicates a breakdown of immunological tolerance to vascular antigens, similar to the systemic lupus erythematosus (SLE)‐like condition observed in RASGRP1‐deficient mice [9, 16]. Individuals with RASGRP1 deficiency may benefit from regular coagulation screening, especially in the presence of unexplained neurological or vascular symptoms.

The vulnerability to EBV‐derived malignancies, especially HL and DLBCL, stems from a distinct deficiency in antigen‐induced T‐cell proliferation [17, 18]. RASGRP1 is essential for connecting TCR signaling to the RAS‐MAPK/ERK pathway [11, 14]. Its absence prevents the upregulation of CTP synthetase 1 and the CD27–CD70 axis, incapacitating the host's ability to manage primary EBV infection [14, 18].

Finally, our analysis highlights a pronounced survival disparity based on treatment. Patients treated conservatively with immunosuppressants experienced a 45% mortality rate attributable to refractory malignancy or severe infection [3, 7, 10, 11]. In contrast, all patients who received HSCT attained remission with a 100% survival rate. These data support early HSCT as the definitive care instead of a salvage therapy. In summary, early genetic diagnosis, screening for coagulopathy, and timely referral for HSCT are crucial to reduce the life‐threatening risks linked with RASGRP1 deficiency.

## Author Contributions

Conceptualization: Nima Parvaneh. Methodology: Kosar Asna Ashari, Bahareh Yaghmaie, Neda Pak, Mohammad Shahrooei, Vahid Ziaee, and Nima Parvaneh. Formal analysis and investigation: Kosar Asna Ashari, Bahareh Yaghmaie, Neda Pak, Mohammad Shahrooei, Vahid Ziaee, and Nima Parvaneh. Writing – original draft preparation: Kosar Asna Ashari and Nima Parvaneh. Writing – review and editing: Kosar Asna Ashari and Nima Parvaneh. Supervision: Nima Parvaneh.

## Funding

The authors have nothing to report.

## Ethics Statement

Informed consent for participating in the study and publication of data was obtained from the parents. Our study adhered to the Declaration of Helsinki.

## Conflicts of Interest

The authors declare no conflicts of interest.

## Supporting information



Supporting File 1

Supporting File 2

## Data Availability

The identified variants have been submitted to ClinVar database (SUB15839731 and SUB15839887) and are currently undergoing review.
