# Supplementary material for: RASGRP1 Deficiency Manifesting as Severe Vasculopathy and Fatal Autoimmune Hemolytic Anemia
Source: EJHaem. 2026 May 29;7(3):e70275. doi: 10.1002/jha2.70275 (PMC13240410; doi:10.1002/jha2.70275)
Supplement: Supplementary file 1 — Supporting File 1 [file JHA2-7-e70275-s001.docx]

|  | **First admission** | **Second admission** | **Normal ranges** |
| --- | --- | --- | --- |
| **WBC (cells/μL)** | 1480 | 2920 | 4000-10000 |
| **Hb (g/dL)** | 7.6 | 3.2 | Age dependent |
| **Platelets (×10^9^/L )** | 16 | 107 | 150-450 |
| **Neutrophils (cells/μL)** | 810 | 1830 | 2000-7000 |
| **Lymphocytes (cells/μL)** | 580 | 710 | 800-4000 |
| **CD3+ (cells/μL)** | 460 | - | 1400-3700 |
| **CD4+ (cells/μL)** | 118 | - | 700-2200 |
| **CD8+ (cells/μL)** | 342 | - | 490-1300 |
| **CD19+ (cells/μL)** | 225 | - | 390-1400 |
| **CD56+ (cells/μL)** | 86 | - | 130-720 |
| **IgG (mg/dL)** | 1634 | - | 386-1470 |
| **IgM (mg/dL)** | 198 | - | 37-224 |
| **IgA (mg/dL)** | 192 | - | 25-154 |
| **Anti-tetanus IgG (IU/mL)** | 0.7 | - | <0.1 Insufficient |
| **Anti-diphtheria IgG (IU/mL)** | 0.2 | - | <0.1 Insufficient |
| **ESR-1hr (mm/hr)** | 56 | 130 | 0-10 |
| **CRP (mg/L)** | 128 | 75 | Positive>6 |
| **Protein C (%)** | 78 | - | 25-140 |
| **Protein S (%)** | 75 | - | 15-130 |
| **Anti-thrombin III (%)** | 99 | - | 90-120 |
| **BUN (mg/dl)** | 5 | 11 | 5-18 |
| **Creatinine (mg/dl)** | 0.5 | 0.3 | 0.4-0.65 |
| **AST (U/L)** | 49 | 24 | 10-40 |
| **ALT(U/L)** | 38 | 86 | 10-40 |
| **FANA** | 1/80 (fine speckled) | - | Positive>1/80 |
| **Anti dS-DNA (IU/ml)** | 1 | - | Positive>25 |
| **Anti B2 Glycoprotein IgM (U/ml)** | 200 | - | Positive>18 |
| **Anti B2 Glycoprotein IgG (U/ml)** | 25.9 | - | Positive>18 |
| **Anti cardiolipin IgM (U/ml)** | 29.5 | - | Positive>18 |
| **Anti cardiolipin IgG (U/ml)** | 101.1 | - | Positive>18 |
| **Coombs direct** | Negative | Positive | - |
| **Coombs indirect** | Negative | Positive | - |
| **Retic count (%)** | 6.1 | 7.9 | 0.5-1.5 |
| **Ferritin (ng/ml)** | 226 | - | 10-60 |
| **EBV viral load** | Undetectable | Undetectable | - |
| **COVID-19 PCR** | Negative | Negative | - |
| **HIV Ab** | 0.1 | - | <1.0 non-reactive |
| **Blood culture** | Negative | Negative | - |

WBC: white blood cells; Hb: hemoglobin; CD: cluster of differentiation; IgG: immunoglobulin; ESR-1hr: erythrocyte sedimentation rate at 1 hour; CRP: C-reactive protein; BUN: blood urea nitrogen; Cr, creatinine; AST: aspartate aminotransferase; ALT: alanine aminotransferase; FANA: fluorescent antinuclear antibody; dsDNA: double-stranded DNA antibody; EBV: Epstein–Barr virus; HIV: human immunodeficiency virus
