# Supplementary material for: RASGRP1 Deficiency Manifesting as Severe Vasculopathy and Fatal Autoimmune Hemolytic Anemia
Source: EJHaem. 2026 May 29;7(3):e70275. doi: 10.1002/jha2.70275 (PMC13240410; doi:10.1002/jha2.70275)
Supplement: Supplementary file 2 — Supporting File 2 [file JHA2-7-e70275-s002.docx]

|  | **P1** | **P2** | **P3** | **P4** | **P5** | **P6** | **P7** | **P8** | **P9** | **P10** | **P11** | **P12** | **P13** | **P14** | **P15 (Our case)** |
| --- | --- | --- | --- | --- | --- | --- | --- | --- | --- | --- | --- | --- | --- | --- | --- |
| **Gender** | Male | Female | Female | Male | Male | Female | Female | Male | Male | Female | Female | Male | Male | Female | Male |
| **Ethnicity** | Turkish | Iraqi | - | - | - | - | Turkish | Palestinian | Palestinian | Iranian | Iranian | Lebanese | Iranian | Malay | Iranian |
| **Consanguinity** | Yes | Yes | No | No | Yes | Yes | Yes | Yes | Yes | Yes | Yes | Yes | Yes | Yes | Yes |
| **Clinical presentations** | | | | | | | | | | | | | | | |
| **Hematologic** |  | AIHA, ITP | AIHA, ITP | AIHA |  |  |  | AIHA, ITP, TTP | AIHA, ITP |  | ITP | AIHA, ITP | AIHA |  | AIHA |
| **Lymphomas** | None | DLBCL  EBV | None | None | Hodgkin  EBV | Hodgkin  EBV | DLBCL  EBV | None | DLBCL  EBV | Hodgkin  EBV | Hodgkin  EBV | None | None | Hodgkin  EBV | None |
| **Autoantibodies** |  |  | ANA anti-SSA, Coombs, aTPO,  anti-TG | ANA, anti-C3d, anti-IgG |  |  |  | Coombs, anti-β2gp | ANA , Coombs |  | ASMA, ANA | Coombs |  | Coombs, ANA  , anti-dsDNA | Coombs,  Lupus anticoagulant, anti-β2gp, anti-cardiolipin, anti-phospholipid |
| **Organomegaly/LAP** |  | Splenomegaly, LAP | Hepatosplenomegaly, LAP | Hepatosplenomegaly, LAP | Hepatosplenomegaly, LAP |  | Hepatosplenomegaly, LAP | Hepatosplenomegaly, LAP | Hepatosplenomegaly, LAP | Hepatosplenomegaly | Splenomegaly |  |  | Hepatosplenomegaly, LAP |  |
| **Infections** | Pneumonias, bronchiectasis, upper respiratory tract infection, HSV | Ear infections, skin abscesses | Pneumonias | Pneumonias, CMV, Aspergillosis,  axillary LN TB | Disseminated TB | Pneumocystis jirovecii pneumonia | Pneumonia, Herpes zoster, molluscum contagiosum | Recurrent pneumonia, dental abscess, CMV | Recurrent pneumonia, CMV, HSV1, | Recurrent upper respiratory infections | Recurrent sino-pulmonary infections, lung abscess, esophageal candidiasis, CMV | Upper respiratory tract and skin infections | Recurrent AOM | Recurrent pneumonia, recurrent AOM | Varicella Zoster |
| **Dermatologic** |  | EV |  |  |  |  |  |  |  |  |  |  |  |  | Bullous pemphigoid |
| **GI** | FTT | Chronic diarrhea, FTT |  |  |  |  | FTT | FTT, steatorrhea | FTT, autoimmune hepatitis |  | Autoimmune hepatitis | Early onset colitis |  | FTT |  |
| **Other** |  |  | Leiomyoma | HLH |  |  | Fatigue |  | Severe posterior uveitis |  | SNHL | Thrombosis |  | Infantile nephrotic syndrome | Stroke, DVT |
| **Immunologic profile** | | | | | | | | | | | | | | | |
| **CD4+ T cells** | Low | Low | Normal | Low | Low | Low | Low | Low | Low | Low | Low | Low |  | Low | Low |
| **CD8+ T cells** | High | High | High | High | Low | Low | High | High | High | Normal | High | Low |  | Low | Low |
| **B cells** | Low | Low | Normal | Normal | Low | Low | Normal | Normal | Normal | Normal | Normal | Normal |  | Low | Normal |
| **IgG** | Normal | Low | High | High | Normal | Normal | Normal | Normal | Normal | Low | Normal | Normal |  | High | Normal |
| **IgM** | Normal | High | High | High | Normal | Normal | Normal | Normal | Normal | Normal | Normal | Normal |  | High | Normal |
| **T cell proliferation response to mitogens and antigens** | Low | Low |  |  | Low | Low |  |  |  | Low | Low |  |  |  |  |
| **NK cell number** | Normal | Normal | Low | Normal | Low | Low | Normal | Normal | Normal | Normal | Normal | Normal |  | High | Normal |
| **EBV status** | Positive | Positive | Negative | Positive | Positive | Positive | Positive | Positive | Positive | Positive | Positive | Negative | Negative | Positive | Negative |
| **RASGRP1 Mutation** | Hom  c.726C>T,  p. Arg246*  Exon 9 | Hom c.771G>A;p.Trp257*  Exon 10 | Compound Het c.641C>T, c.964A>T  p.Thr214Ile, p.Lys322*  Exon 9, Exon 11 | Compound Het c.641C>T, c.964A>T  p.Thr214Ile, p.Lys322*  Exon 9, Exon 11 | Hom c.1910_1911insAG, p.Ala638Glyfs*16  Exon 16 | Hom c.1910_1911insAG, p.Ala638Glyfs*16  Exon 16 | Hom c.649_650inv, p.Glu217Arg  Exon 9 | Hom c.1111_1114del, p.Asp371Ilefs*7  Exon 11 | Hom c.1111_1114del, p.Asp371Ilefs*7  Exon 11 | Hom c.1242 G > A, p. Thr414=  Exon 12 | Hom c.811 C > T p. Arg271*  Exon 10 | Hom c.1396_1397InsA, p.Thr466Asnfs*3  Exon 11 | c.934A>G, p.Thr312Ala  Exon 11 | Hom c.2180G>A, p.Trp727*  Exon 16 | Hom c.1720+1G>A and c.1720+2T>C  Intron 13 |
| **Treatment** | Monthly IVIG, prophylactic AB,  chemotherapy for lymphoma, HSCT | IVIG, prophylactic AB, rituximab,  chemotherapy | IVIG, steroid | IVIG, mycophenolate mofetil | Chemotherapy, autologous HSCT, rituximab | Chemotherapy | Chemotherapy, rituximab, HSCT | IVIG, steroid | IVIG, steroid, chemotherapy, intravitreal methotrexate | Unknown | Unknown | IVIG, steroid, mycophenolate mofetil, rituximab | ABs | Steroid, rapamycin, chemotherapy, ABs, rituximab, HSCT | IVIG, steroid, eculizumab, sirolimus, cyclosporine, bortezomib, plasmapheresis |
| **Outcome** | Remission | Deceased (complications of lymphoma) | Remission | Remission | Remission | Deceased (relapse of Hodgkin lymphoma) | Remission | Deceased (TTP) | Remission | Deceased (lymphoma) | Remission | Remission | Unknown | Remission | Deceased (anemia) |
| **Ref** | Salzer E , et al. 2016 | Platt CD, et al. 2017 | Mao H, et al. 2018 | Mao H, et al. 2018 | Winter S, et al. 2018 | Winter S, et al. 2018 | Somekh I, et al. 2018 | Somekh I, et al. 2018 | Somekh I, et al. 2018 | Momenilandi M, et al. 2022 | Momenilandi M, et al. 2022 | Mansour R, et al. 2023 | Jamee M, et al. 2024 | Nashrudin KNM, et al.2025 | Current study |

AIHA: autoimmune hemolytic anemia; ITP: immune thrombocytopenic purpura; ANA: antinuclear antibody; SSA: Sjogren’s-syndrome-related antigen A autoantibodies; aTPO: thyroid peroxidase antibodies; anti-TG: anti-thyroglobulin antibodies; Ig: immunoglobulin; ASMA: anti smooth muscle antibody; anti-DsDNA: anti-double-stranded deoxyribonucleic acid antibodies; LAP: lymphadenopathy; EBV: Epstein-Barr virus; LN: lymph node; TB: Tuberculosis; CMV: Cytomegalovirus; HSV: Herpes simplex virus; AOM: acute otitis media; FTT: failure to thrive; HLH: hemophagocytic lymphohistiocytosis; SNHL: sensorineural hearing loss; DLBL: diffuse large B-cell lymphoma; BM: bone marrow; IVIG: intravenous immunoglobulin ; AB: antibiotic ; HSCT: hematopoietic stem cell transplantation; TTP: thrombotic thrombocytopenia purpura
